# Efficacy of Lateral Neck Plain Radiographs in Suspecting an Obstruction at the Base of the Tongue in Children With Sleep Disordered Breathing

**DOI:** 10.7759/cureus.98771

**Published:** 2025-12-08

**Authors:** Nourhan Howidi, Marwah Al Shmanee, Ajay P Dsouza, Mohamad A Bitar

**Affiliations:** 1 Graduate Medical Education, Dubai Health, Dubai, ARE; 2 Diagnostic Imaging, Al Jalila Children's Specialty Hospital, Dubai, ARE; 3 Pediatric Otolaryngology, Al Jalila Children's Specialty Hospital, Dubai, ARE

**Keywords:** airway obstruction, base of tongue, diagnostic, lingual tonsil, sleep endoscopy, x-ray

## Abstract

Background

Obstructed breathing in children often requires adenotonsillar surgery as the primary treatment. Persistent symptoms may necessitate further diagnostic investigations and surgeries. Base of tongue (BOT) enlargement or lingual tonsillar hypertrophy (LTH), a common source of obstruction in these cases, is detected during flexible endoscopy, usually under sedation in the operating theatre. The role of lateral neck plain radiographs (LNPR) in this context is unknown.

Objectives

This study aims to assess the effectiveness of LNPR in diagnosing or suspecting obstruction at the level of BOT in the pediatric age group.

Methods

A retrospective cohort was conducted on children with upper airway obstruction between January 2020 and December 2023, following either failed adenotonsillar surgery or with unremarkable physical examination findings. Data included demographics, symptoms, comorbidities, sleep endoscopy findings, surgeries, and outcomes. The LNPRs were analyzed blindly by a radiologist using study-specific measurements. Radiological findings were compared with intraoperative findings, including those of sleep endoscopy, to assess diagnostic accuracy, specifically the ability to distinguish LTH from BOT enlargement and correlate with surgical outcomes.

Results

A total of 22 patients were included. Among them, 68.2% (15/22) were male, while 31.8% (7/22) were female. The mean age of the patients was 7.3 years, with a median of 6.2 years. Of these, 45.5% (10/22) had prior surgeries. Sleep endoscopy was done in 81.8% (18/22), and polysomnography in 40.9% (9/22). LNPRs accurately predicted obstruction at the BOT in 87.5% (14/16) of cases, with 72.2% (13/18) agreement between radiological and intraoperative findings regarding BOT surface description.

Conclusion

The LNPR is a valuable adjunct in evaluating BOT obstruction and may guide surgical planning. Standardization of radiological measures is recommended.

## Introduction

Sleep disordered breathing (SDB) in children is often caused by obstruction at multiple levels. In the existing literature, the primary treatment for children with SDB is adenotonsillar surgery. Those who fail this procedure and show persistent symptoms are more likely to undergo drug-induced sleep endoscopy (DISE) that will guide the need for additional surgical intervention, which often addresses lingual tonsillar hypertrophy (LTH), enlargement of lymphoid tissue, and/or base of tongue enlargement (BOTE), enlargement of tongue base bulk not limited to lymphoid tissue [1,2]. However, whether this can be detected or at least suspected through a simple radiological examination, eliminating the need for a diagnostic procedure requiring a general anesthetic, remains uncertain.

It is estimated that 80% of children diagnosed with obstructive sleep apnea (OSA) experience successful resolution of their condition through adenotonsillar surgery [3]. Those who fail this procedure can have other contributing factors, such as laryngomalacia, craniofacial disproportion, oropharyngeal soft tissue redundancy linked to obesity, macroglossia, glossoptosis, large BOT, or LTH [4].

The diagnostic assessment is meant to pinpoint specific sites of obstruction through evaluations conducted both while the individual is awake and asleep. Common diagnostic modalities during wakefulness include flexible laryngoscopy and lateral neck plain radiographs (LNPR), whereas assessments during a sleep-induced state include DISE and cine magnetic resonance imaging [1]. The LNPR might be effective in identifying LTH, especially in children with conditions such as Down syndrome or obesity. Radiographically identifiable lingual tonsil tissue is observed in 30% of children, with a slightly higher percentage (34%) detected in the Down syndrome population [1]. Compared to other methods, it has advantages such as ease of administration, low cost, and good clinical correlation [1,3].

Surgical management due to obstruction at the level of BOT should be guided by the diagnostic findings. Treatment modalities include lingual tonsillectomy, posterior midline glossectomy (PMG), tongue suspension suture, tongue-lip adhesion, hyoid suspension, and hypoglossal nerve stimulation [5,6]. Lingual tonsillectomy addresses LTH. It is generally safe, with readmission and postoperative bleeding rates comparable to those of adenotonsillectomy [3]. Posterior midline glossectomy is performed with or without lingual tonsillectomy, resulting in the removal of all tissues contributing to the obstruction at the level of BOT. It is thought that resurfacing the lingual tonsil tissue along with PMG can result in more uniform scarring of the tongue base, which is hypothesized to improve outcomes [4].

Our study aims to analyze the diagnostic ability of LNPR in identifying obstructions at the level of BOT in children who have failed previous upper airway surgery or those whose initial physical examination did not correlate with the severity of their presenting symptoms. A secondary objective is to determine if LNPR can distinguish between LTH and BOTE.

## Materials and methods

Study design

We conducted a retrospective cohort study that evaluated children with SDB who underwent LNPR between January 2020 and December 2023. The images were performed either following unsuccessful adenoid and/or tonsillar surgery or as part of an initial diagnostic assessment. The study population consisted of children aged 18 years or younger. Individuals with a history of previous BOT surgery were excluded from the study.

Data collection

Relevant clinical and demographic data, including LNPR, were retrospectively extracted from the electronic medical records (EMRs) of patients under the care of the senior author during the study period. Variables of interest included patient demographics (gender, age at first presentation), presenting symptoms, physical examination findings, comorbidities, and findings from sleep endoscopy, when available. Surgical and procedural interventions undertaken by the patients were reviewed, along with follow-up documentation to evaluate the occurrence of any complications or adverse events. A senior radiologist blindly examined all LNPRs without access to the patients’ clinical details to ensure objective interpretation.

Radiological measurements

**Figure 1:**
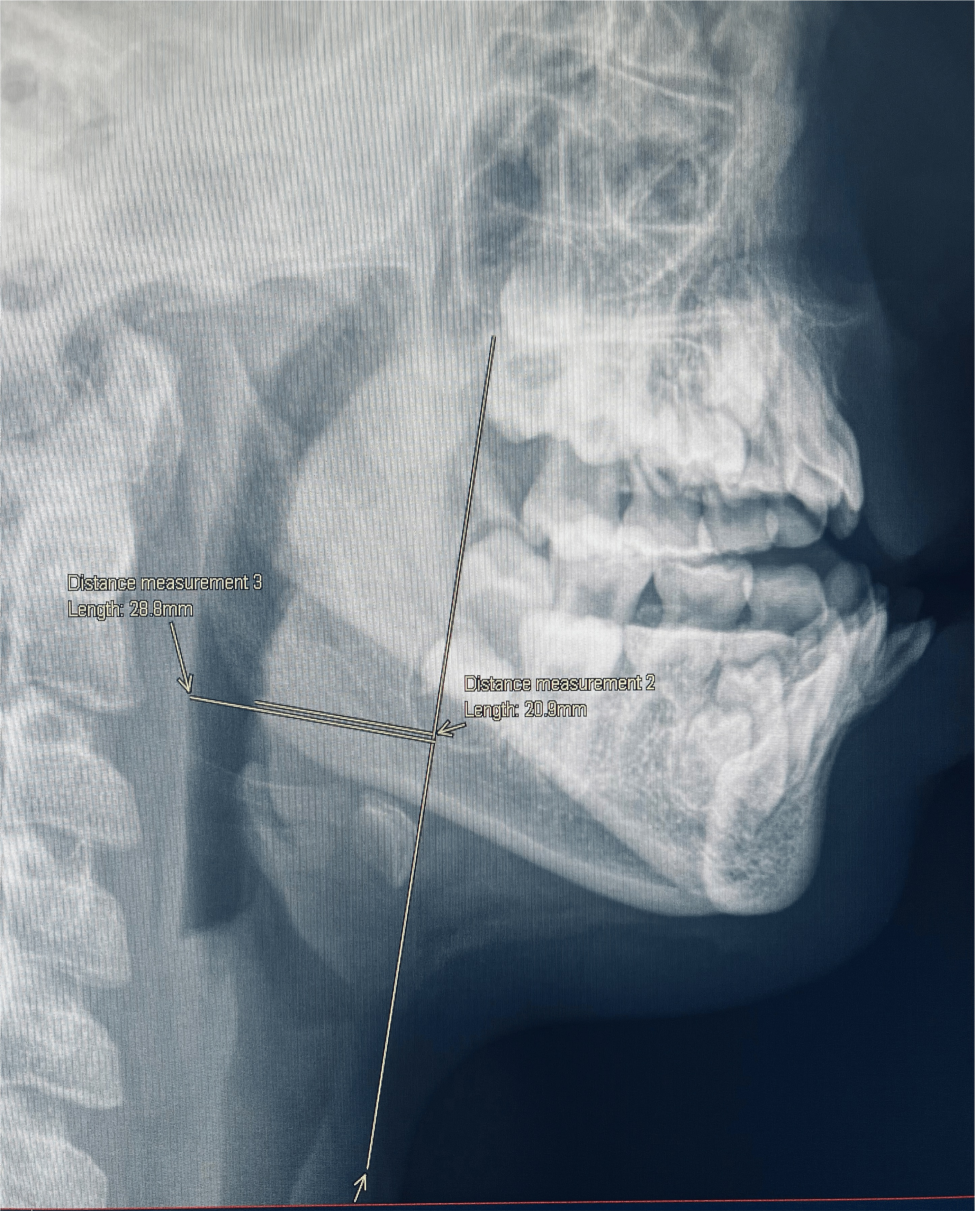
X-ray demonstrating the objective measurement of the BOT A vertical line (baseline) is drawn from the posterior bony edge of the hard palate tangentially over the anterior surface of the hyoid bone. The hypopharyngeal airway (A): line extending from the posterior pharyngeal wall to the baseline. The thickness of BOT (B): line from maximum convexity to baseline. BOT: base of tongue

Radiological measurements were performed using images retrieved from the institutional picture archiving and communication system (PACS). Standard lateral radiographs of the neck were used, with windowing adjusted to provide optimal visualization of soft tissue. All images were carefully assessed to exclude rotation or improper positioning of the neck. Only true lateral views were included for measurement. Quantitative assessments were conducted on high-resolution PACS monitors using integrated digital calipers and standard measurement tools available within the PACS system. On each radiograph, a vertical reference line was drawn from the posterior bony edge of the hard palate, tangentially extending along the anterior surface of the hyoid bone; this served as the baseline, referred to as the HH line, as shown in Figure 1. Two perpendicular measurements were taken above the level of the hyoid bone. The first measurement (A) extended from the posterior pharyngeal wall to the HH line, intersecting the point of maximum soft tissue density on the radiograph. This distance represented the combined thickness of the base of the tongue and the hypopharyngeal airway. The second measurement (B) was drawn from the anterior limit of this soft tissue opacity (corresponding to the base of the tongue) to the HH line. A ratio was then derived by dividing B by A and multiplying by 100, thereby calculating the percentage of hypopharyngeal airway encroachment by the base of the tongue (Figure 1).

**Figure 2:**
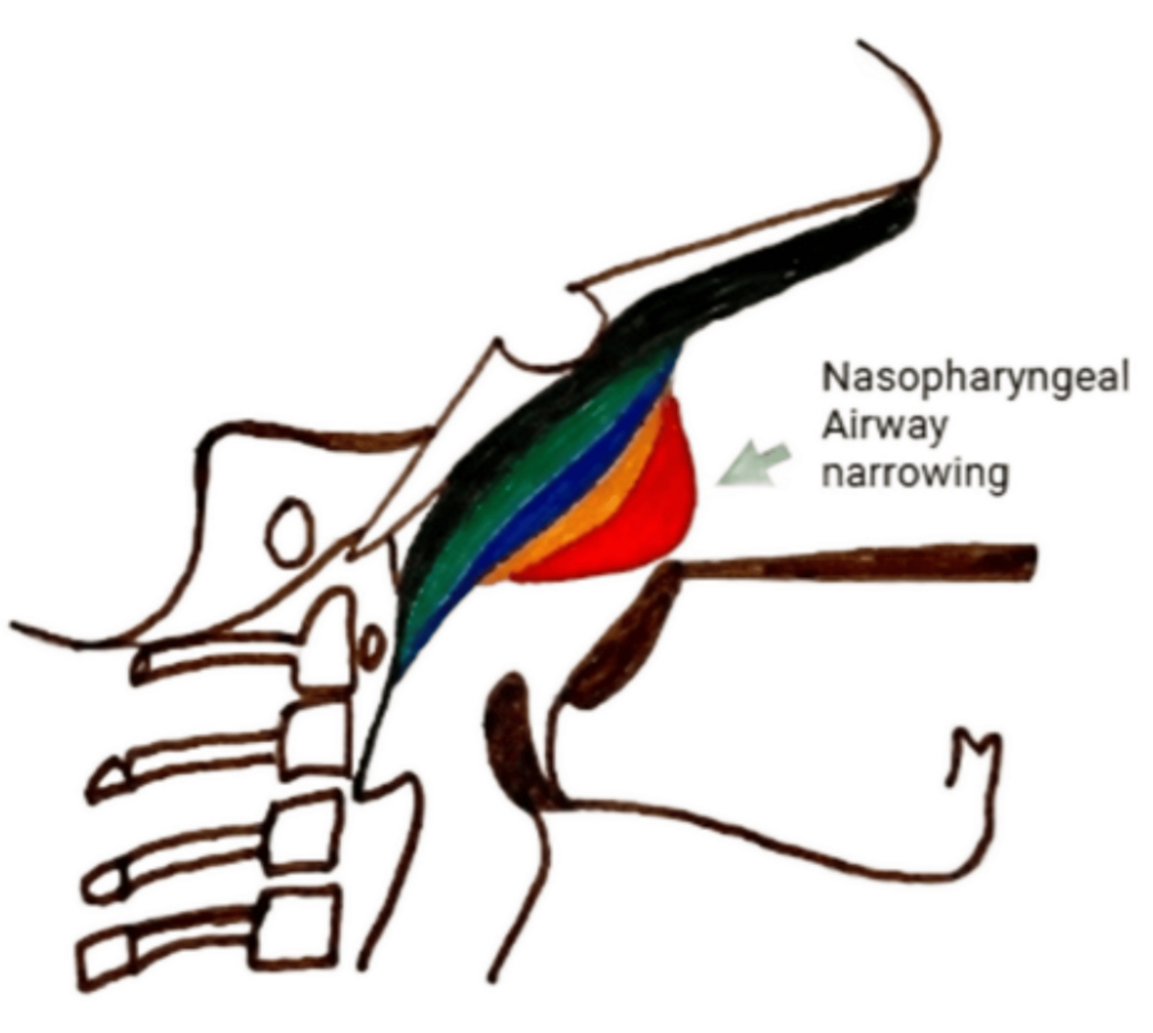
Schematic diagram of lateral neck X-ray for grading of adenoid enlargement Grade 0 (Green): Concave soft tissue; no mass effect; Grade 1 (Blue): Convex adenoids; indentation without airway narrowing; Grade 2 (Orange): Convex adenoids; indentation with partial airway narrowing; Grade 3 (Red): Convex adenoids; complete airway obliteration Image credits: Dr. Ajay P. Dsouza

In addition to these linear measurements, qualitative assessments of tonsillar and adenoidal soft tissue prominence were performed using grading systems, as shown below, which were specifically developed for this study. These grading scales were formulated to provide a reproducible method for evaluating soft tissue prominence on lateral neck radiographs. The tonsillar shadow grading is classified into three levels (Figure 2). In Grade 0, the tonsillar shadow is not seen. In Grade 1, the tonsillar shadow is visible but does not reach the tip of the epiglottis, and there is narrowing of the oropharyngeal air column. In Grade 2, the tonsillar shadow reaches the epiglottis and extends beyond it. The adenoid grading is divided into four levels. In Grade 0, there is a concave contour of the nasopharyngeal soft tissue with no mass effect. In Grade 1, the adenoids appear convex with indentation but without narrowing of the nasopharyngeal airway. In Grade 2, the adenoids are convex with indentation and partial narrowing of the nasopharyngeal airway. In Grade 3, the adenoids are convex with complete obliteration of the nasopharyngeal air column.

Data management and ethical considerations

All study-related data were securely stored in a restricted-access facility, accessible only to the Primary Investigator and Co-Investigators. Electronic records, including data collection forms and analysis files, were maintained within a secure intranet folder. Patient identifiers were excluded, and the analysis was performed on de-identified datasets to ensure confidentiality and data protection. The study involved no direct patient interaction, as data were derived from pre-existing EMRs, minimizing additional risks to participants. The study was approved by the Institutional Review Board (IRB) of Mohammed bin Rashid University (MBRU) and the Dubai Scientific Research and Ethics Committee (DSREC) at the Dubai Health Authority (DHA). It adhered to ethical guidelines to ensure no harm to participants. The IRB approval number is MBRU IRB-2023-341.

Statistical analysis

Data analysis was conducted using basic descriptive statistics to summarize patient demographics and clinical characteristics. Continuous variables were reported as mean, median, and range, while categorical variables were described as frequencies and percentages. Radiological findings, particularly those related to the BOT, were reviewed to assess airway obstruction, with obstruction greater than 50% classified as a positive finding. The LNPR interpretations were compared with intraoperative findings to evaluate the diagnostic accuracy of the imaging. Subgroup analyses were performed to explore potential associations. Diagnostic agreement between LNPR measurements and intraoperative findings was quantified as the overall percentage of concordant cases. No inferential statistical tests were applied due to the limited sample size (n = 22); therefore, all results are descriptive and exploratory in nature. The results were organized and presented in tables and graphical formats using Microsoft Excel (Microsoft Corporation, Redmond, WA, US), with additional findings included to provide a comprehensive interpretation of the clinical data.

## Results

A total of 22 patients were included in the study. Of these, 68.2% (15/22) were male, and 31.8% (7/22) were female. The age distribution showed that 63.6% (14/22) of the patients were older than 5 years. The mean age was 7.34 years, with a median of 6.25 years. The age ranged from 2.1 to 15.1 years, with an overall range of 13.0 years. Regarding surgical history, 45.5% (10/22) of the patients had previously undergone surgery but experienced treatment failure, while 54.5% (12/22) were de novo cases. Comorbidities were identified in 68.2% (15/22) of the patients. Polysomnography (PSG) was performed in 40.9% (9/22) of cases, while 81.8% (18/22) underwent sleep endoscopy. The surgical plan was determined based on the intraoperative findings, primarily from the results of sleep endoscopy, and procedures were conducted simultaneously.

**Figure 3:**
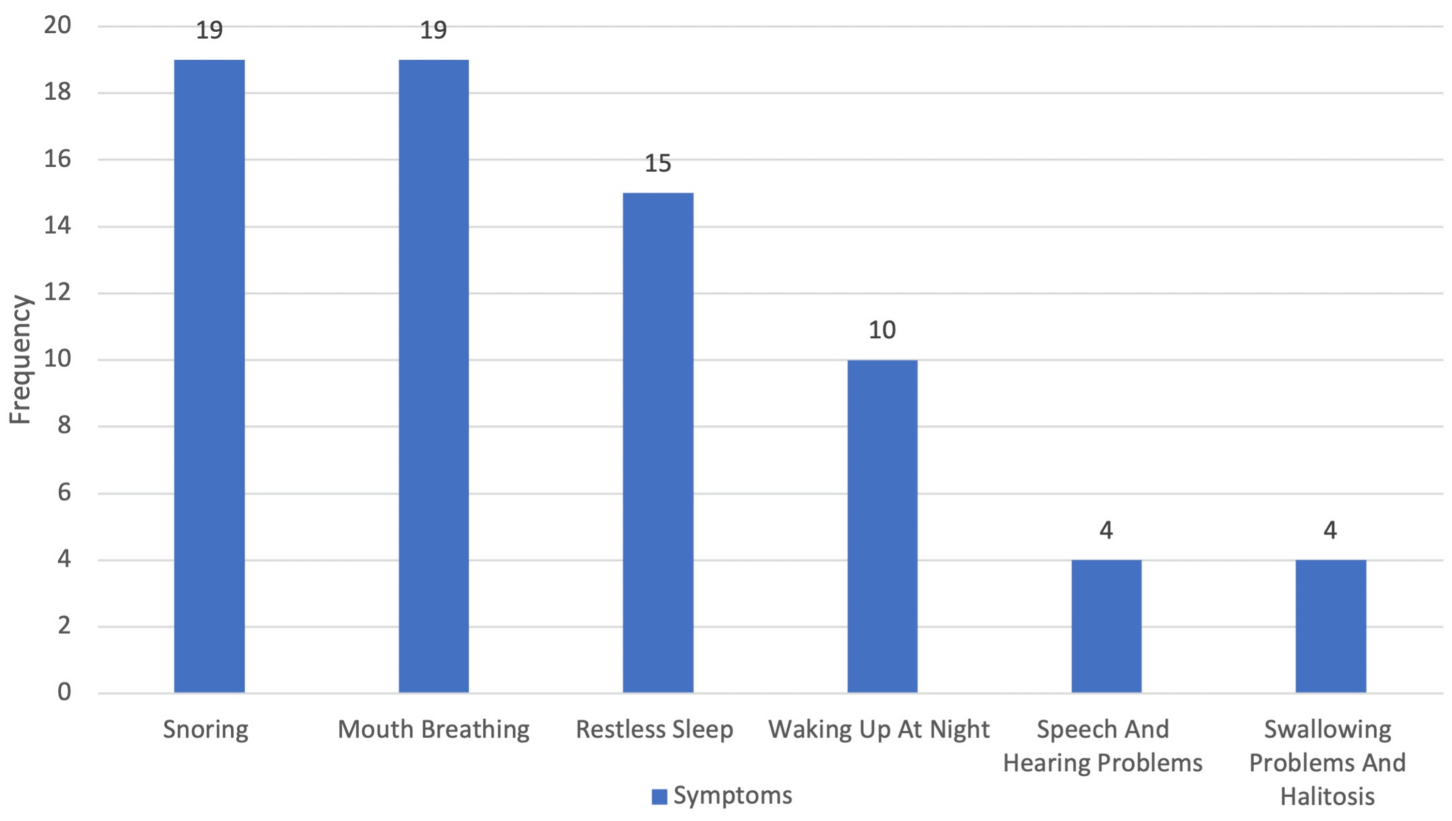
Presenting symptoms of enrolled patients

The most frequently reported symptoms were snoring (86.4%, 19/22) and mouth breathing (86.4%, 19/22), followed by restless sleep (68.2%, 15/22) and night awakenings (45.5%, 10/22). Other reported symptoms include speech and hearing difficulties, swallowing problems, and halitosis (Figure 3).

**Figure 4:**
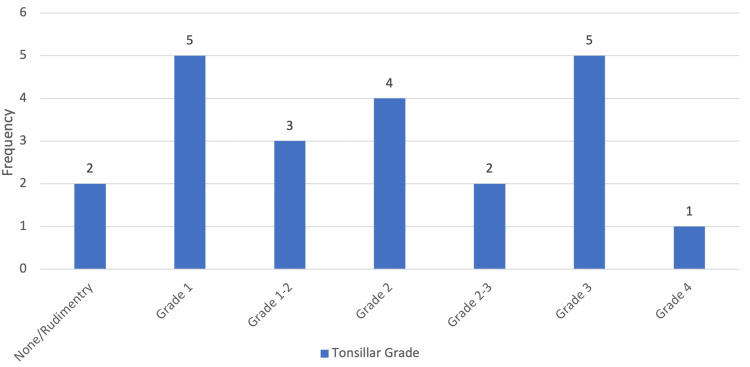
Degree of tonsil size as seen on physical examination

On physical examination, the size of the tonsils varied among patients, with most having tonsils classified as Grade 1 to Grade 3. A minority had either rudimentary/nonexistent tonsils or Grade 4 enlargement. The distribution of tonsil sizes is illustrated in Figure 4.

Obstruction at the level of the BOT was described on LNPR as a percentage of encroachment using the measurements explained in Figure 1 and compared to the intraoperative findings in Table 1. An obstruction of more than 50% was considered positive. Twenty patients had LNPR suitable for proper measurements; two were not adequate (view not optimal), with one showing a grossly significant obstruction, while the other did not. Fourteen out of 16 measurements (87.5%) showed positive correlation with the intraoperative findings. The LNPRs were also inspected to determine if they could distinguish between LTH and BOT obstruction. The radiological description of the BOT surface was consistent with the intraoperative findings in 72.2% (13/18) of the patients, as described in Table 1.

**Table 1:** Radiological vs intra-operative findings Co-M: co-morbidities; T&A: adenotonsillectomy; Adx: adenoidectomy; LT: lingual tonsillectomy; T: tonsils; OSA: obstructive sleep apnea; SDB: sleep disordered breathing; DS: Down syndrome; GH: growth hormone; GDD: global developmental delay; VCP: vocal cords paralysis

#	Age (y)	Previous Surgery	Co-M / Diagnosis	Tonsil Grade	Sleep Endoscopy		Surgery Performed	Findings of Lateral Neck X-Ray	
					BOT	LTH		BOT Obstruction/ Appearance	BOT Objective Measurement
1	6.00	T&A reduction	Drooling / OSA	G2		Yes	LT + T&A reduction	Irregular BOT	16.4/27.6 = 59.4%
2	3.00	Adx	GERD / OSA	G3		Yes	LT + T reduction	Smooth BOT	Grossly no
3	15.00	-	SDB	G3		Yes	LT + T&A reduction	Smooth BOT	28.6/37.5 = 76.2%
4	3.50	Posterior cordotomy	VCP / SDB	G3		Yes	LT + T&A reduction	Irregular BOT	8/29.4 = 27%
5	4.75	T&A reduction	GDD / OSA	G0		Yes	LT + Adx	Irregular BOT	8.4/25 = 33%
6	6.50	-	OSA	G1	Yes	Yes	LT + Adx	Smooth BOT	17.1/24.3 = 70.3%
7	14.50	Posterior cordotomy	VCP / SDB	G1	Yes	Yes	LT	Smooth BOT	Grossly obstructive
8	4.00	-	GERD/ Asthma/ OSA	G1	Yes	Yes	LT + Adx	Smooth BOT	17.7/28.7 = 61%
9	11.00	Adx	SDB	G2	Yes		LT + BOT reduction	Smooth BOT	23/28 = 82%
10	6.00	Adx	DS / SDB	G4		Yes	LT + T reduction	Irregular BOT	15.5/28.1 = 55%
11	2.11	Adx	SDB	G2	Yes		LT	Smooth BOT	14.4/23.4 =61%
12	2.40	T&A reduction	GH deficiency / OSA	G3		Yes	LT	Irregular BOT	16.5/24.2 = 68%
13	7.00	-	SDB	G1		Yes	LT	Irregular BOT	23.3/27.2 = 85%
14	9.10	-	SDB	G2	Yes		LT + BOT reduction	Smooth BOT	19.2/29.3 = 65%
15	15.11	-	SDB	G1	Yes	Yes	LT + BOT reduction	Irregular BOT	21.9/28.9 = 75%
16	8.00	-	DS / OSA	G2		Yes	LT	Irregular BOT	15.9/26.6 = 59.7%
17	8.40	T&A	DS / OSA	G0	Yes	Yes	LT + BOT reduction	Irregular BOT	20.9/28.8 = 72%
18	11.00	-	Autism / SDB	G2		Yes	LT + T&A reduction	Smooth BOT	17.1/29.1 = 58.7%
19	4.00	-	SDB	G3	planned			Irregular BOT	13.9/28.8 = 48%
20	5.00	-	SDB	G2	planned			Smooth BOT	15.9/26 = 61.1%
21	9.00	-	SDB	G2	planned			Irregular BOT	20.7/31.8 = 65%
22	6.00	-	Autism / SDB	G2	planned			Irregular BOT	17.1/29.8 = 57%

## Discussion

We evaluated the utility of LNPR in predicting BOT obstruction in a selected pediatric population with SDB or OSA. Our findings suggest that the LNPR and the introduced measurement tools applied to it show agreement with the intraoperative findings for assessing airway obstruction at the level of the BOT and guiding surgical decision-making and counseling parents.

Our study population had an age distribution skewed toward older children, reflecting the fact that such patients either failed a previous surgery at a younger age or presented at an age where we do not expect obstruction, at least at the level of the adenoids. Comorbidities were present in 68.2% (15/22) of the cohort, indicating the complexity of the affected patients and the need for multiple assessment methods.

X-rays, particularly LNPR, are widely used in clinical practice due to their accessibility, relatively low cost, and ability to provide rapid diagnostic information, making them a valuable first-line imaging tool in many clinical settings. The LNPR has traditionally been employed as an initial imaging technique for assessing upper airway obstruction in children [7,8]. It has proven particularly useful in evaluating conditions such as adenoid hypertrophy, epiglottitis, and abnormalities of the retropharyngeal space [9,10]. However, its role in detecting BOT obstruction has been less extensively explored. Our study investigated its utility in this context, especially in patients who fail an initial surgery to relieve the upper airway obstruction. However, we extended that to patients whose physical examination did not explain the presenting symptoms.

While advanced imaging modalities, such as dynamic MRI, offer detailed anatomical insights, they are resource-intensive and often necessitate sedation [10]. In contrast, LNPR provides a practical alternative for initial evaluation. The positive correlation observed in our study between LNPR findings and intraoperative observations suggests its potential in estimating the narrowing at the level of BOT. Furthermore, our findings indicate that LNPR may be able to distinguish between BOT enlargement and LTH in a significant proportion of cases, highlighting its potential role in preoperative planning. While some studies have questioned the sensitivity of static imaging in capturing dynamic airway conditions, our results suggest that, when interpreted in conjunction with existing clinical symptoms and physical examination, LNPR can provide meaningful insights into obstructive patterns, particularly in settings where more advanced modalities are unavailable [9].

The LNPR measurements showed agreement with intraoperative observations in 87.5% (14/16) of cases, indicating a high level of concordance for detecting obstruction at the level of the BOT. Furthermore, LNPR positively distinguished BOTE from LTH in 72.2% (13/18) of cases. These findings suggest that LNPR could serve as a non-invasive adjunct to current diagnostic tools, aiding in early identification of candidates for BOT reduction or LTH ablation [11]. This makes them a potential tool in refining preoperative planning and facilitating informed discussions with parents regarding the need for more extensive airway interventions [12]. Additionally, LNPR may serve as a reliable screening tool in settings where advanced imaging or sleep endoscopy is not readily available [13,14].

Despite the promising findings, several limitations must be acknowledged. First, the study population was highly selective, comprising patients with persistent symptoms despite previous interventions or those whose symptoms did not align with the clinical examination. This introduces a potential selection bias, limiting the generalizability of our results. It is not clear if the measurements would detect obstruction at the level of the BOT in surgically naïve children with the presence of large tonsils and adenoids, which already explain the symptoms, particularly the high-risk patients, like those with Down syndrome and obese patients. Second, while LNPR measurements correlated well with intraoperative findings, the absence of a control group prevents definitive conclusions regarding the specificity and sensitivity of LNPR in a broader pediatric population. Future studies should focus on validating these findings in larger, more diverse cohorts, including children with varying degrees of airway obstruction and those without symptoms, to further confirm the results. However, it will be challenging to find enough LNPRs that were performed in asymptomatic patients. Establishing standardized measurement criteria for LNPR would enhance its diagnostic accuracy and facilitate broader clinical adoption.

## Conclusions

The suggested measurements on LNPR showed that it can be a valuable tool for predicting obstruction at the level of the BOT. Its use can aid in guiding the surgeon to plan for the possible need for LTH ablation and/or BOT reduction surgery, as well as in preoperative counseling of parents regarding these procedures. Further validation through studies involving a “normal” control group is essential to establish standardized measurement criteria and enhance diagnostic accuracy.
